# The differential impact of COVID-19 on mental health: Implications of ethnicity, sexual orientation, and disability status in the United States

**DOI:** 10.3389/fpsyg.2022.902094

**Published:** 2022-09-13

**Authors:** Jordan M. Brooks, Cyrano Patton, Sharon Maroukel, Amy M. Perez, Liya Levanda

**Affiliations:** California School of Professional Psychology, Alliant International University, San Francisco, CA, United States

**Keywords:** COVID-19, pandemic, depression, anxiety, minorities

## Abstract

The COVID-19 pandemic’s effects on mental health interact with preexisting health risks and disparities to impact varying populations differently. This study explored the relationship between demographic variables (e.g., ethnicity, sexual orientation, and disability status), distress and mental health (e.g., depression, anxiety, somatic complaints, and pandemic distress), and vulnerability factors for COVID-19 (e.g., personal health vulnerabilities, community members’ health vulnerabilities, and environmental exposure risks at work or home). An online cross-sectional study was conducted from 18 June to 17 July 2020, reflecting the impact of early phase COVID-19 pandemic and related shelter-in-place measures in the United States. Participants were adults residing in the United States (*N* = 594), with substantial subsamples (*N* ≥ 70) of American Indian, Asian American, African-American, and Hispanic and/or Latinx participants, as well as people with disabilities and sexual minorities. Outcomes measured were depression, hopelessness, somatic complaints, anxiety-related disorders, locus of control (LOC), and a novel measure of pandemic-related distress. Data were analyzed using analyses of covariance (ANCOVA), chi-square test, and correlation coefficients. Generally, younger individuals, and those with less financial power—across all identities—suffered more distress. When controlling for age, lower financial power was associated with higher scores on the Center for Epidemiologic Studies Depression Scale-Revised (CESD-R; *r* = –0.21, *p* = < 0.001), Beck Hopelessness Scale (BHS; *r* = –0.17, *p* < 0.001), Patient Health Questionnaire-15 (PHQ-15; *r* = –0.09, *p* = 0.01), Screen for Child Anxiety Related Emotional Disorders for Adults Panic Disorder (SCARED-A PD; *r* = –0.14, *p* < 0.001), SCARED-A generalized anxiety disorder (GAD; *r* = –0.13, *p* = 0.002), SCARED-A obsessive–compulsive disorder (OCD; *r* = –0.08, *p* = 0.04), and the COVID-19 Pandemic Distress restriction/disconnection scale (C19PDS; *r* = –0.10, *p* = 0.009). In addition, disparities were found, in general, for marginalized identities by gender, sexual orientation, and disability status. Importantly, each ethnicity subsample showed a unique pattern of relationships between COVID-19 risk variables and mental health symptoms. The results support the hypothesis that any pandemic may amplify preexisting social and financial disparities. Overall, interventions at the clinical, governmental, or health equity level should take into consideration the needs of vulnerable groups.

## Introduction

At the end of 2019 and early months of 2020, the severe acute respiratory syndrome coronavirus 2 (SARS-CoV-2) spread throughout China and subsequently throughout the world. The SARS-CoV-2 infection—causing the coronavirus disease 19 (COVID-19)—spread rapidly, compelling countries and global governing bodies to enact preventative efforts such as shelter-in-place or quarantine mandates, that disrupted normal ways of life. Early on, healthcare and other essential workers were identified as particularly at risk for not only infection by SARS-CoV-2, but also for mental health distress (Lai et al., 2020). In early 2020, China identified mental health concerns in the general population as well, citing panic, anxiety, and depression as major concerns (Qiu et al., 2020). Actual illness, income and job inequality, governmental preparation and communication, and stigma toward those infected have been cited as concerns for mental health (Graham et al., 2020; Hossain et al., 2021; Piltch-Loeb et al., 2021). In the United States, of particular concern were loneliness, low distress tolerance, and COVID-19 worry, which are associated with clinical symptoms of depression, anxiety, and post-traumatic stress disorder (PTSD; Liu et al., 2020). A metareview of quarantine or shelter-in-place measures in historical pandemics and epidemics identified negative psychological effects including post-traumatic stress, confusion, and anger (Brooks et al., 2020).

Individuals with marginalized identities experienced disparities in health and other life outcomes prior to the COVID-19 pandemic, many of which are exacerbated in the face of pandemic or other disasters, especially in the realm of healthcare, and particularly for those with multiple minority identities (Gray et al., 2020). Kline (2020) notes that health inequity should be at the forefront of conversations surrounding global responses to the COVID-19 pandemic, specifically higher risk populations, such as elderly adults and people who have marginalized identities, but it is unclear whether those are the greatest risk for infection and mortality are also at greatest risk for psychological distress.

Research continues to emphasize racial and ethnic disparities in COVID-19 cases, where people of racial minority groups are overrepresented (for review, see Pan et al., 2020; Sze et al., 2020). Furthermore, a review of COVID-19 literature found that over half of the manuscripts from the first wave of COVID-19 did not report racial distribution (Raghav et al., 2021). While minority groups make up small percentages of the overall population in the United States, recent studies show that within their individual racial and ethnic categories, Hispanic/Latinx, American Indian/Alaskan Native, Native Hawaiian/Pacific Islanders, and Black individuals are disproportionately infected with SARS-CoV-2 (Raine et al., 2020). Black and African-American people, for example, are contracting the disease and dying from it at higher rates than other ethnic groups (Raine et al., 2020; Thebault et al., 2020). Both race and socioeconomic status (SES) have been identified as long-standing factors of poorer health outcomes and early mortality (Chu et al., 2007). Gelaye et al. (2020) proposed that stress-associated neurobiological activity is much higher for Black, Indigenous, and People of Color (BIPOC), and this chronic elevation predisposes these individuals to chronic stress, under-activated antiviral pathway genes, and therefore higher likelihood of contracting and experiencing worse outcomes with diseases such as COVID-19.

African-American and Latinx people experienced significant spikes in unemployment as a result of the pandemic (Couch et al., 2020), followed by Asian Americans, even when holding equivalent education to their counterparts of any other race, likely a factor of the growing racism and xenophobia associated with the virus (Kim et al., 2021). While this trend of economic shock for ethnic minorities dates back to the Great Depression, Asian Americans have also experienced an increased prevalence of hate crimes related to the pandemic (Stop AAPI Hate, 2021).

The primary concern during COVID-19 for lesbian, gay, bisexual, and queer (LGBQ) sexual minorities, and their gender minority constituents, is an exacerbated effect of already immense struggles related to mental health, such as suicidal ideation and family rejection (Salerno et al., 2020a). Although most of the general population reported mental health or physical health struggles related to the COVID-19 pandemic, gender and sexual minority (GSM) individuals are affected in unique ways, including reporting higher rates of substance use, domestic abuse, discrimination, COVID-19 worry, grief, disconnection/isolation, depression, and anxiety compared to their sexual majority counterparts (e.g., LGBT Foundation, 2020; Peterson et al., 2020; Kamal et al., 2021).

Gender and sexual minority populations already experience systemic oppression that limits access to necessary resources such as lodging, education, and healthcare, as well as socially supportive networks that would otherwise buffer negative mental health outcomes (Gibb et al., 2020). The social distancing and stay-at-home restrictions exacerbate this limitation greatly. The GSM community already has disproportionate rates of poverty (Badgett et al., 2020) and twice the average rate of service-industry employment, unemployment, or employment requiring high traffic social contact, which is each of particular concern during the pandemic (Salerno et al., 2020b). Loss of or lack of healthcare is a related concern for GSM individuals, especially for the transgender and gender expansive (TGE) community, who face immense financial disparity and discrimination in healthcare (Safer et al., 2016). All in all, the available literature on GSM considerations during the COVID-19 pandemic advocates for accessible and effective resources and supports to bolster equity for the GSM community (Signorelli et al., 2020).

The COVID-19 pandemic has also profoundly affected people with disabilities, a population that makes up about 26% of adults in the United States based on definitions by the Americans with Disabilities Act (ADA). Reviews of safety measures and accessibility services from educational systems, news, or governmental reports noted a dearth of disaster planning or considerations for individuals with disabilities, as well as a lack of sufficient information and resources or aid (Jesus et al., 2021). People with disabilities have been impacted by the COVID-19 pandemic in three main areas: higher risk of infections, loss of services and accessibility, and deficit or denial of care through measures such as medical rationing (Lund et al., 2020).

While only some ADA disabilities increase risk of infection by SARS-CoV-2 (e.g., *via* cardiovascular risk; Kamalakannan et al., 2021), many carry risk factors for greater repercussions from infection (Boyle et al., 2020). Abedi et al. (2020) noted a connection between higher disability rates, higher poverty rates, and higher COVID-19 mortality rates by county, proposing that decreased mobility and access to healthcare might explain the higher mortality rate.

National and global measures to prevent the spread of SARS-CoV-2 themselves impacted disabled individuals and their families’ ways of life, requiring significant changes in services or routines, including loss of educational, behavioral, or healthcare supports (Jesus et al., 2021). Individuals receiving assistance from caregivers, aides, or providers may be forced to choose between receiving aid and following shelter-in-place protocols or have physical limitations that make certain precautions difficult (World Health Organization, 2020). As school or work endeavors have shifted toward online platforms, many disabled individuals’ typical resources have been rendered obsolete or unavailable, especially for those in rural or low-income areas (Lund et al., 2020).

One of the largest issues impacting the disabled community and the current COVID-19 pandemic is the implementation of medical rationing of medical supplies across the world. Although medical rationing has been used historically, the current societal context warrants concerns about ethical implementation and has caused many disability activists to speak out against the discriminatory practice due to its ableist mentality that those with disabilities and older age are more disposable than younger, non-disabled individuals which, in some instances, may prevent individuals with disabilities the proper or even life-sustaining care needed (Boyle et al., 2020; Lund and Ayers, 2020). Andrews et al. (2020, p. 7) emphasize the American Psychological Association’s stance that “a disabled person’s ability to achieve their goals depends less on the nature of disability and individual coping skills than on personal, familial, and systemic interactions with schools, employers, healthcare providers, and communities.”

Well-documented historical disparities for marginalized groups in the United States highlight the importance of studying the relationship between COVID-19 risk factors, mental health outcomes, and demographic factors including sexual orientation, disability status, and a range of ethnicities. This study examines a broad set of mental health-related outcomes including depression, anxiety, hopelessness, somatic symptoms, LOC, and pandemic distress. This study addresses gaps in the literature by examining these outcomes comprehensively across multiple marginalized identities to identify their unique experiences and health inequity.

## Materials and methods

### Participants

The study included a nationally representative sample (*N* = 594; with missing data minimum *N* = 490) of adult participants (ages 18–78, *M* = 35.00; *SD* = 15.42). Inclusion criteria included those who were at least 18 years old, currently living in the United States, and who had the ability and access to complete an online survey in the English language. Of the 50 states, Hawaii, Maine, and North Dakota did not have participants, as well as no one from the District of Colombia or Puerto Rico. A power analysis was conducted based on the most rigorous planned analysis for the data (MANOVA), with a minimum of 98 participants; the sample goal was inflated to a minimum of 120 to account for any invalid or missing data. The majority of participants were female (65%), resided in dense urban areas (36%), and reported ongoing but loosening shelter-in-place orders in their county at the time of data collection (50%). Economic status was calculated by where participants fell in relation to the poverty line, based on their reported income and the number of people supported by that income. Table 1 provides descriptive statistics for the overall sample’s demographic variables, along with those of our sexual minority and ADA-disability subsamples. Of our ADA-disability sample, 56% had physical disabilities, 14% had vision-related disabilities, and 11% were hard of hearing.

**TABLE 1:** Participant demographics and key variables.

	Overall sample *N* = 594	Sexual minority subsample *N* = 95	ADA disability subsample *N* = 88
Characteristic	*n* (%)	*n* (%)	*n* (%)
**Age**			
*18 to 29* *30 to 39* *40 to 49* *50 and above*	201 (34) 163 (28) 88 (15) 142 (24)	43 (45) 28 (30) 7 (7) 17 (18)	15 (17) 20 (23) 17 (19) 36 (41)
**Gender**			
*Male* *Female* *Non-binary or transgender*	203 (34) 385 (65) 6 (1)	26 (27) 64 (67) 5 (5)	29 (33) 59 (67) 0 (0)
**Ethnicity**			
*American Indian or Alaskan Native* *Asian or Asian American* *African American* *European American* *Hispanic or Latinx* *Other/Multiethnic/Did not answer*	70 (12) 111 (19) 88 (15) 208 (35) 81 (14) 36 (6)	12 (13) 8 (8) 12 (13) 41 (43) 13 (14) 9 (10)	12 (14) 10 (11) 22 (25) 29 (33) 11 (13) 4 (5)
**Population Density**			
*Rural* *Suburban* *Urban* *Did not answer*	119 (20) 199 (34) 213 (36) 63 (11)	17 (18) 29 (31) 35 (37) 14 (15)	15 (17) 24 (27) 40 (46) 9 (10)
**Education**			
*Less than a bachelor’s degree* *Bachelor’s degree* *Graduate degree*	303 (51) 175 (30) 116 (20)	53 (56) 24 (25) 18 (19)	56 (64) 19 (22) 13 (15)
**Local Shelter-in-Place Status**			
*Ongoing* *Ongoing but loosened* *Has ended* *Never*	121 (20) 299 (50) 108 (18) 66 (11)	27 (28) 13 (14) 7 (7) 48 (51)	21 (24) 16 (18) 5 (6) 46 (52)
**Sexual Orientation**			
*Heterosexual*	499 (84)	0 (0)	71 (81)
*Lesbian*	11 (2)	11 (12)	2 (2)
*Gay*	18 (3)	18 (19)	4 (5)
*Bisexual*	41 (7)	41 (43)	7 (8)
*Queer*	13 (2)	13 (14)	3 (3)
*Prefer not to say*	12 (2)	12 (13)	1 (1)
**Disability Status**			
*No*	506 (85)	78 (82)	0 (0)
*Yes*	88 (15)	17 (18)	88 (100)
**Economic Status**			
*Below poverty line*	110 (19)	15 (16)	30 (34)
*At poverty line (1x)*	121 (20)	31 (33)	23 (26)
*Above poverty line*	117 (20)	23 (25)	13 (15)
*At Living Wage (2x)*	132 (22)	20 (21)	12 (14)
*Middle or high class (3x*+)	107 (18)	5 (5)	10 (11)

### Procedure

Quota convenience sampling, a type of stratified sampling, was utilized to recruit participants until adequate statistical representation (at least 70 participants) was attained across a number of demographic categories: ethnicity (American Indians, African Americans, Asian Americans, European Americans, and Hispanic/Latinx individuals); gender (cisgender men and women); sexual orientation (sexual minorities and heterosexual individuals), and urbanicity (rural, suburban, and urban). This study does not claim to have an exhaustive sample for these groups nor is it meant to represent the present prevalence rates of the U.S. population across demographic categories. The survey was hosted by Qualtrics, using a combination of researcher-initiated advertisement, and Qualtrics’ own online recruitment, with up to $3.00 compensation for completed surveys. Through Qualtrics, simple logic quota was used to meet the identified minimum 70 participants for each of the demographic groups above; this minimum was determined adequate utilizing power analysis for planned analyses. Participant data were collected from 18 June to 17 July 2020.

Participants provided informed consent electronically, were given a list of mental health and COVID-19 resources, then took 20–27 min to complete the online survey. The full survey gathered information on demographics, coping skills and their efficacy, psychological and physiological distress, and pandemic-specific experiences and risks, with question formats including Likert’s scale, multiple choice, and free response (measures utilized for the present analyses discussed in detail below). This study was approved by the Alliant International University Institutional Review Board (IRB; protocol #2004176143, approved 10 June 2020).

### Measures

#### Demographics

Demographic items queried ethnicity, gender, sexual orientation, disability status, education, employment, income, and living situation, among other factors. For vulnerabilities to COVID-19, participants identified whether they had (1) personal chronic health issues related to COVID-19 risk, such as being immunocompromised or having lung or heart trouble; (2) family, community members, or clients through work had chronic health issues related to COVID-19 risk; or (3) home or work environments that place them at increased risk for exposure to COVID-19 (i.e., due to flow of customers, work in a healthcare setting, or housemates who neglect safety protocols).

#### Depression

The Center for Epidemiologic Studies Depression Scale-Revised Short Form (CES-D-R 10; α > 0.86; Miller et al., 2008) is a 10-item version of the full CESD-R and a well-validated measure of depression (Van Dam and Earleywine, 2011), with higher scores indicating more frequent symptoms of depression. Participants rate how often they experience depressive symptoms on a four-point scale between “Rarely or None of the Time” (less than 1 day) and “All of the Time” (5–7 days).

#### Hopelessness

The Beck Hopelessness Scale (BHS; Beck et al., 1974) is a 20-item true–false questionnaire, with higher scores representing higher levels of hopelessness. Prior research has deemed the BHS as both reliable (α = 0.88) and valid in undergraduate college populations (Steed, 2001). This study utilized the most predictive four items out of the original 20, as recommended by Aish et al. (2001) and further confirmed by Yip and Cheung (2006).

#### Anxiety

The Screen for Child Anxiety Related Emotional Disorders for Adults (SCARED-A; van Steensel and Bögels, 2014) is a 71-item measure evaluating anxiety in adults and has nine subscales that correspond with anxiety-related diagnoses in the DSM-IV, four of which are utilized in the present analyses: generalized anxiety disorder (GAD; 9 items; α = 0.84, –0.89), social anxiety disorder (SAD; 9 items; α = 0.83, –0.90), panic disorder (PD; 13 items; α = 0.80, –0.86), and obsessive–compulsive disorder (OCD; 9 items; α = 0.62, –0.76). The GAD subscale focuses on various sources of worry, while the PD subscale focuses on situations that may cause fear and panic. The SAD subscale includes items such as “I feel nervous when I go to a party.” The OCD subscale includes items on ruminations and behaviors, such as “I want things to be in a fixed order.” Higher scores indicate greater frequency of symptoms described on a three-point scale (0 = Almost Never, 1 = Sometimes, and 2 = Often), to which a clinical cutoff score can be applied.

#### Somatization

The Patient Health Questionnaire-15 (PHQ-15; α = 0.80; Kroenke et al., 2002) is a 15-item questionnaire evaluating the severity of somatic symptoms, with higher scores indicating greater severity in more areas of discomfort. Participants rate the degree to which they have been bothered by each symptom (e.g., “stomach pain” and “dizziness”) during the past 7 days on a three-point scale from “Not Bothered at All” to “Bothered a Lot.” The PHQ-15 demonstrated good reliability and validity in adult primary care and other samples (Kroenke et al., 2002).

#### Distress

The COVID-19 Pandemic Distress Scale (C19PDS; Chang et al., 2021) is a novel, self-authored, self-report measure of distress regarding a variety of facets of the pandemic with shelter-in-place protocols. The C19PDS has 19 items (Full Scale *a* = 0.93), twelve measuring distress due to disconnection and restriction of freedom (Disconnection/Restriction, *a* = 0.92; e.g., “Loneliness,” “Missing my typical hobbies and exercise”) and six measuring distress due to fears around sickness and uncertainty (Fear/Uncertainty, *a* = 0.88; e.g., “The uncertainty of it all,” “The idea of being contaminated and getting sick”), on a five-point Likert Scale of 0 (Not at All Bothered) to 4 (Incredibly Bothered). The full C19PDS and the disconnection/restriction scale have excellent internal reliability, while the fear/uncertainty scales have good internal reliability. The total scale demonstrated strong convergent validity *via* correlation with the Perceived Stress Scale (PSS-10; Cohen et al., 1983).

#### Locus of control

Locus of control was measured by a single item seven-point scale that asked subjects, “How much control do you feel you have over your own life?” from 1 (No Control) to 7 (Complete Control), with higher scores indicating greater internal LOC and lower scores indicating more external LOC. There is evidence that a single item measure is a valid alternative brief measure of LOC as evidenced by Bugaighis and Schumm (1983) and Kovaleva (2012).

### Statistical approach

All data were analyzed using IBM SPSS Version 27. All assumptions for analyses were examined and met. Correlations examined relationships between demographic, COVID-19 risk variables, and mental health outcomes. One-tailed correlations were considered to evaluate statistical significance due to our directional hypotheses that marginalized identities would be positively correlated with measures of psychological distress. Five additional sets of partial correlations were conducted to examine relationship between COVID-19 risk and mental health outcomes within ethnicity subsamples.

Chi-square analyses examined rates of COVID-19 risk for the demographic variables of ethnicity, sexual orientation, and disability. One-way ANCOVAs compared ethnicity, disability status, and sexual orientation, controlling for the effect of age, with Bonferroni *post hoc* tests conducted as needed, due to the variability in sample sizes. Exploratory ANCOVA was conducted for gender.

## Results

**FIGURE 1:**
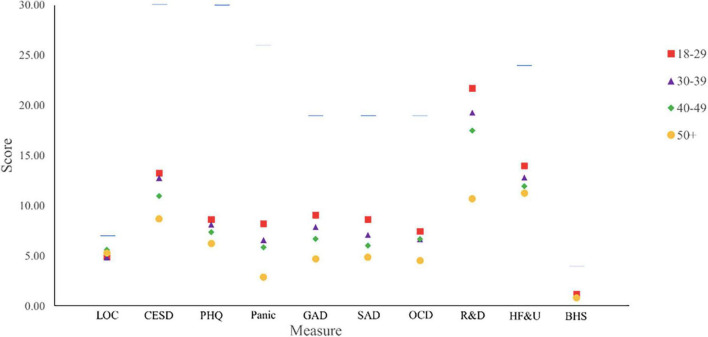
Average scores on mental health measures by age group. Figure displays maximum score for each measure with horizontal line. The maximum score for the R&D subscale is 44 and is not shown due to space. LOC, Locus of Control scale; CESD, The Center for Epidemiologic Studies Depression Scale-Revised Short Form; PHQ, Patient HealthQ uestionnaire-15; Panic, Panic subscale of the SCARED-A; GAD, Generalized Anxiety subscale of the SCARED-A; SAD, Social Anxiety subscale of the SCARED-A; OCD, Obsessive Compulsive subscale of the SCARED-A; R&D, Restriction and Disconnection subscale of the C19PDS; HF&U, Health Fears and Uncertainty subscale of the C19PDS; BHS, Beck Hopelessness Scale.

Partial correlations controlling for the effect of age were run between all suitable study variables (see Table 2), because bivariate correlations revealed age as a significant correlate to many variables, including every mental health variable (see Figure 1 and Table 2, Column 1).

**TABLE 2:** Partial correlation matrix controlling for age.

Variable	1§	2	3	4	5	6	7	8	9	10	11	12	13	14	15	16	17	18	19	20	21	22	23	24	25
1. Age	1.00																								
2. Cismale Gender	0.13**	1.00																							
3. Sexual Minority	–0.10*	–0.05	1.00																						
4. ADA Disability	0.17†	–0.02	0.02	1.00																					
5. Financial Power	0.08	0.14†	–0.13**	–0.10**	1.00																				
6. African American	–0.06	0.05	–0.01	0.15†	–0.07	1.00																			
7. American Indian	0.01	–0.01	0.05	0.04	–0.12**	–0.13†	1.00																		
8. Asian American	–0.17†	–0.02	–0.13†	–0.09*	0.09*	–0.22†	–0.20†	1.00																	
9. European American	0.28†	0.03	0.12**	–0.07*	0.11**	–0.32†	–0.26†	–0.32†	1.00																
10. Hispanic/Latinx	–0.17†	–0.05	0.01	0.01	–0.08*	–0.15†	–0.16†	–0.23†	–0.25†	1.00															
11. CV Risk: Self	0.31†	–0.04	0.08*	0.21†	–0.11**	–0.04	0.12**	0.04	–0.03	–0.04	1.00														
12. CV Risk: Community	–0.07	–0.06	0.10**	0.05	0.03	–0.03	–0.01	–0.12**	0.17†	–0.02	0.00	1.00													
13. CV Risk: Environmental	–0.24†	0.12**	–0.02	0.03	–0.01	0.02	–0.01	–0.05	0.04	0.02	0.06	0.05	1.00												
14. Sheltering-in-Place	0.01	0.14†	0.90*	0.07	0.08*	0.02	0.00	0.02	0.03	–0.07	0.05	–0.14†	0.06	1.00											
15. Urbanicity	–0.01	0.20†	0.03	0.09*	0.08*	0.06	–0.08*	–0.01	0.01	0.07	–0.01	0.03	–0.02	0.17†	1.00										
16. Depression (CESD)	–0.28†	–0.12**	0.08*	0.10**	–0.21†	–0.03	–0.03	–0.05	0.14†	–0.04	0.11**	0.17†	0.07*	–0.06	–0.01	1.00									
17. Hopelessness (BHS)	–0.10*	–0.07	0.13**	0.09*	–0.17†	–0.03	–0.01	–0.03	0.08*	0.04	0.06	0.10*	0.05	–0.06	–0.02	0.47†	1.00								
18. Somatic Symptoms (PHQ)	–0.17†	–0.13**	0.06	0.22†	–0.09*	–0.01	0.03	–0.05	0.04	–0.02	0.12**	0.12**	0.12**	0.03	0.00	0.50†	0.24†	1.00							
19. PD Scale (SCARED-A)	–0.35†	–0.02	–0.01	0.12**	–0.14†	–0.01	–0.04	0.02	0.03	0.01	0.11**	0.03	0.16†	0.04	0.04	0.58†	0.32†	0.56†	1.00						
20. GAD Scale (SCARED-A)	–0.36†	–0.11**	0.08*	0.09*	–0.13**	–0.10**	–0.02	–0.01	0.18†	–0.05	0.09*	0.11**	0.03	0.00	–0.01	0.60†	0.35†	0.48†	0.65†	1.00					
21. SAD Scale (SCARED-A)	–0.32†	–0.07	0.06	0.10*	–0.06	–0.10**	0.01	0.04	0.04	0.00	0.09*	0.04	0.01	0.03	–0.01	0.47†	0.34†	0.43†	0.64†	0.71†	1.00				
22. OCD Scale (SCARED-A)	–0.31†	0.03	0.02	0.11**	–0.08*	0.04	0.02	0.03	–0.02	–0.03	0.06	0.03	0.09*	0.01	0.05	0.46†	0.24†	0.44†	0.72†	0.66†	0.63†	1.00			
23. Pandemic Distress (R/D)	–0.42†	0.04	–0.01	0.07	–0.10**	0.01	–0.03	0.01	0.04	0.01	0.04	0.04	0.05	0.15†	0.15†	0.38†	0.19†	0.30†	0.43†	0.32†	0.24†	0.40†	1.00		
24. Pandemic Distress (HF/U)	–0.17†	–0.13†	0.04	0.09*	–0.07	–0.02	0.00	0.01	0.05	0.03	0.13†	0.13**	0.00	0.02	0.02	0.44†	0.22†	0.33†	0.33†	0.46†	0.35†	0.37†	0.52†	1.00	
25. LOC	0.14†	0.18†	–0.10*	–0.02	0.25†	0.09*	0.04	–0.03	–0.09*	–0.08*	–0.07	–0.12**	0.05	0.08*	0.02	–0.44†	–0.43†	–0.19†	–0.23†	–0.34†	–0.30†	–0.12**	–0.14†	–0.18†	1.00

*p < 0.05; **p < 0.01; †*p* < 0.001.

^§^ Bivariate correlations between age and other variables, demonstrating why it was controlled for in the overall partial correlation matrix.

One-tailed; *N* = 490–592; positive correlations with gender mean scores are higher for cisgender men than combined cisgender female and TGE sample; finance is an ordinal variable based on household income per household/family size; positive correlations with urbanicity mean scores are higher for city dwellers than suburban or rural dwellers. R/D and HF/U are subscales of the CV19PDS (see section Materials and Methods).

LOC, locus of control.

### Ethnicity

#### Ethnicity and COVID-19 risk

Chi-square analyses revealed that rates of personal health-related vulnerabilities to COVID-19 significantly differed by ethnicity (see Table 3). One highlight of *post hoc* analyses revealed significant differences with the American Indian sample endorsing higher personal health-related vulnerabilities to COVID-19 compared with all other ethnicity categories. The rate of health-related vulnerabilities to COVID-19 in one’s home, family, or work community also significantly differed by ethnicity (Table 3). *Post hoc* analyses revealed the European American sample endorsed higher community health-related vulnerabilities than the American Indian, African-American, and Asian American samples. Environment-related vulnerabilities to COVID-19 at home and/or work did not significantly vary by ethnicity, despite a trend in the current literature for a higher percentage of the Hispanic/Latinx participants to endorse environmental exposure risks to SARS-CoV-2 during the pandemic (McNicholas and Poydock, 2020; Rogers et al., 2020).

**TABLE 3:** Chi-square of ethnicity, gender, sexual orientation, disability status, and COVID-19 risk.

		Personal health vulnerability	Community: Health vulnerability	Environmental vulnerability
	*N*	%(*n*)	%(*n*)	%(*n*)
*Ethnicity* χ^2^, *df* = 4		15.02**	13.87**	2.50
African American	87	8(7)^ae^	23(20)^b^	41(36)
American Indian	69	25(17)^abcd^	23(16)^a^	36(25)
Asian American	109	12(13)^b^	18(20)^c^	37(41)
European American	208	17(35)^cef^	36(75)^abc^	37(77)
Hispanic/Latinx	81	6(5)^df^	26(21)	46(37)
*Gender* χ^2^, *df* = 1		0.000	2.96	4.31*
Cismale	201	13(27)	23(47)	45(91)
Cisfemale/TGE	389	13(52)	30(117)	36(141)
*Sexual* χ^2^, *df* = 1 *Orientation*		1.27	6.14*	0.04
Heterosexual	496	13(63)	26(128)	39(194)
LGBQ	94	17(16)	38(36)	40(38)
*Disability* χ^2^, *df* = 1		26.67***	2.85	0.003
Yes	88	31(27)	35(31)	39(34)
No	502	10(52)	27(133)	39(197)

*p < 0.05, **p < 0.01, ***p < 0.001.

^a,b,c,d,e^Within each column, pairs of values marked with the same letter are significantly different at the 0.05 level (df = 1); numbers reflect the percentage and total number of individuals in each ethnic group that endorsed each type of risk.

Due to variability in missing data, *N* represents minimum for each analysis set.

#### Ethnicity and mental health outcomes

Analyses of covariance controlling for the effect of age found significant differences by ethnicity on the CESD-R [*F*(4,552) = 2.70, *p* = 0.03], SCARED-A GAD subscale [*F*(4,550) = 5.01, *p* = 0.001], and LOC [*F*(4,492) = 2.79, *p* = 0.03]. The CESD-R and LOC Scales showed no significant differences when evaluated pairwise. However, the European American sample exhibited a greater average depression score (*M* = 12.89; *SE* = 0.46) than other ethnicities by about two points. Similarly, European Americans exhibited the greatest mean GAD score (*M* = 8.39, *SE* = 0.32), significantly greater in pairwise comparisons to both the African-American (*M* = 6.07, *SE* = 0.48, *p* = 0.001) and Hispanic/Latinx samples (*M* = 6.61, *SE* = 0.51, *p* = 0.04). For the LOC scale, African Americans (*M* = 5.50, *SE* = 0.16) exhibited the most internal LOC, closely followed by American Indians (*M* = 5.34, *SE* = 0.15), then Asian Americans (*M* = 5.08, *SE* = 0.16) in the middle, and with the European American (*M* = 4.95, *SE* = 0.12) and Hispanic/Latinx (*M* = 4.89, *SE* = 0.17) samples with the lowest. Finally, each ethnicity subsample demonstrated unique patterns between COVID-19 risk variables and mental health outcomes (Table 4).

**TABLE 4:** Interitem correlations of mental health outcomes and risk by ethnicity group, controlling for age.

Variable	CESD	BHS	PHQ-15	Panic	GAD	SAD	OCD	R&D	HF&U	LoC
**African American (*N* = 88)**										
Personal vulnerability	0.02	0.00	0.21*	0.16	0.07	–0.03	0.14	0.00	0.01	–0.05
Community vulnerability	0.24**	0.05	0.10	0.06	0.23*	0.15	0.22*	0.04	0.22*	–0.11
Environmental exposure	0.04	–0.05	0.09	0.20*	0.06	0.10	0.00	–0.06	–0.10	0.12
Ongoing Shelter-in Place	–0.07	–0.02	0.16	0.06	0.07	0.06	0.01	0.18*	0.01	0.05
Urbanicity	0.14	–0.01	–0.10	–0.04	–0.01	–0.10	0.08	–0.01	0.07	–0.11
**American Indian (*N* = 70)**										
Personal vulnerability	0.16	–0.12	0.30**	0.33**	0.16	0.21*	0.26*	0.08	0.22*	–0.03
Community vulnerability	0.15	0.02	0.30**	0.19	0.27**	0.08	0.15	0.11	0.12	–0.14
Environmental exposure	0.03	0.17	0.17	0.15	0.10	0.19*	0.13	–0.05	–0.06	–0.13
Ongoing Shelter-in Place	–0.15	–0.17	0.03	0.08	–0.05	0.11	0.11	0.13	0.06	0.01
Urbanicity	0.06	0.11	0.20*	0.05	0.06	0.17	0.16	0.25*	0.17	–0.17
**Asian American (*N* = 111)**										
Personal vulnerability	0.02	0.09	0.01	0.04	0.03	0.07	0.07	0.03	0.00	–0.08
Community vulnerability	0.06	0.10	0.12	0.02	0.05	0.01	0.07	0.01	0.06	0.04
Environmental exposure	0.29**	0.20*	0.12	0.24**	0.13	0.03	0.16*	0.25**	0.03	–0.07
Ongoing Shelter-in Place	–0.04	0.03	–0.11	–0.03	–0.03	0.09	0.00	–0.04	0.00	–0.02
Urbanicity	–0.05	–0.11	–0.10	–0.10	–0.12	–0.05	–0.13	0.13	–0.05	–0.02
**European American (*N* = 208)**										
Personal vulnerability	0.15*	0.14*	0.10	0.08	0.14*	0.10	0.02	0.04	0.20**	–0.16**
Community vulnerability	0.17**	0.10	0.11*	–0.02	0.05	–0.05	–0.04	0.02	0.17**	–0.06
Environmental exposure	0.01	–0.03	0.17**	0.21**	0.04	0.04	0.18**	0.03	0.07	0.08
Ongoing Shelter-in Place	–0.11	–0.14*	0.02	0.00	–0.04	–0.05	0.02	0.26†	0.05	0.21**
Urbanicity	–0.08	–0.04	0.04	0.07	–0.02	0.00	0.08	0.21**	–0.06	0.20**
**Hispanic/Latinx (*N* = 81)**										
Personal vulnerability	0.20*	0.05	0.02	0.04	0.00	–0.02	–0.13	–0.02	0.04	0.11
Community vulnerability	0.08	0.10	0.08	–0.06	0.01	0.07	–0.02	0.10	0.12	–0.17
Environmental exposure	–0.02	–0.01	0.11	0.04	–0.16	–0.18	–0.05	–0.01	–0.13	0.18
Ongoing Shelter-in Place	0.18	0.09	0.17	0.15	–0.01	–0.03	–0.11	0.05	–0.12	–0.07
Urbanicity	–0.03	0.06	0.08	0.15	0.08	0.05	0.15	0.17	0.11	0.07

*p < 0.05; **p < 0.01; †*p* < 0.001.

*N* = 558; CESD, The Center for Epidemiologic Studies Depression Scale-Revised Short Form; BHS, Beck Hopelessness Scale; PHQ, Patient Health Questionnaire-15; Panic, Panic subscale of the SCARED-A; GAD, generalized anxiety subscale of the SCARED-A; SAD, social anxiety subscale of the SCARED-A; OCD, obsessive–compulsive subscale of the SCARED-A; R&D, Restriction and Disconnection subscale of the C19PDS; HF&U, Health Fears and Uncertainty subscale of the C19PDS; LOC, Locus of Control Scale.

### Sexual orientation

#### Sexual orientation and COVID-19 risk

Significantly more LGBQ individuals reported health-related vulnerabilities among their own community members, as compared to the heterosexual sample. Personal health-related vulnerabilities or environment-related vulnerabilities to COVID-19 did not vary by sexual orientation (Table 3).

#### Sexual orientation and mental health outcomes

Analyses of covariance examined differences across sexual orientation for ten mental health outcomes, controlling for the effect of age. Scores on the BHS [*F*(1,594) = 9.32, *p* = 0.002], the SCARED-A GAD Scale [*F*(1,589) = 4.00, *p* = 0.046], and LOC scale [*F*(1,552) = 5.10, *p* = 0.021] significantly differed by sexual orientation. The LGBQ sample had the greatest mean BHS score (*M* = 1.36, *SE* = 0.13), in comparison with the heterosexual sample (*M* = 0.93, *SE* = 0.06, *p* = 0.002). Similarly, the LGBQ sample had a higher mean GAD score (*M* = 8.20, *SE* = 0.46) in comparison with the heterosexual sample (*M* = 7.19, *SE* = 0.20, *p* = 0.05). Finally, the heterosexual sample (*M* = 5.17, *SE* = 0.07) exhibited more internal LOC than the LGBQ sample (*M* = 4.76, *SE* = 0.17, *p* = 0.02).

### Disability status

#### Disability status and COVID-19

Participants with disabilities had a significantly higher rate of personal health-related vulnerabilities to COVID-19 than those without (Table 3). Participants did not differ in community or environment-related COVID-19 vulnerabilities by disability status.

#### Disability status and mental health outcomes

Analyses of covariance examined differences between participants with and without disabilities for ten mental health outcomes, controlling for the effect of age. Psychiatric disabilities were excluded to prevent confounds with the mental health outcomes. Participants with physical disabilities had statistically significantly greater scores than non-disabled participants on the CESD-R [*F*(1,578) = 5.74, *p* = 0.017; (*M* = 13.18, *SE* = 0.74; *M* = 11.28, *SE* = 0.28)] and BHS [*F*(1,578) = 4.57, *p* = 0.033; (*M* = 1.26, *SE* = 0.14; *M* = 0.93, *SE* = 0.06)], indicating marginally more frequent symptoms of depression and hopelessness for the disabled population. The only measure in which participants with physical disabilities had both statistically and clinically significant elevations as compared to non-disabled participants was the PHQ-15 [*F*(1,577) = 26.91, *p* < 0.001; (*M* = 10.94, *SE* = 0.66; *M* = 7.18, *SE* = 0.25)]. Participants with physical disabilities also had statistically significantly greater scores than non-disabled participants on the C19PDS Health Fears/Uncertainty Scale [*F*(1,573) = 5.12, *p* = 0.024], and three SCARED-A subscales: GAD [*F*(1,576) = 5.05, *p* = 0.025], Panic Disorder [*F*(1,576) = 7.95, *p* = 0.005], and OCD, [*F*(1,576) = 6.65, *p* = 0.010]. Of these measures, the mean scores between people with and without disabilities differed by one or fewer points on each aforementioned scale.

## Discussion

This study aimed to identify disparities in mental health outcomes related to COVID-19 health risks, ethnicity, sexual orientation, and disability status. This study’s single greatest correlate for distress during early phase COVID-19 was age. The younger the participants, the greater their distress, on average, across all 10 mental health outcomes analyzed, without considering other identities and risk factors, as evidenced by Figure 1. Understanding younger adults’ psychological vulnerability during a pandemic could be useful to the policymakers, physicians, professors, and mental health professionals guiding our population in times such as this, particularly when the natural focus is on protecting older adults with greater COVID-19 risks to physical health.

In terms of interpretation, young people may experience greater suffering around their freedom being restricted, having a smaller community of support to rely on, thwarted desires to date new people, or a lack of resilience derived from surviving previous large-scale national or global crises such as wars, depressions, and epidemics, as compared to their older counterparts. A robust literature on developmental needs and generational influences suggests that young adults, particularly in newer generations, rely more on friendship than familial relations (Levitt et al., 1993) and are less likely to live with their main social supports or consider a partner to be the center of their social universe (Jamieson et al., 2006), while shelter-in-place policies discourage multi-household interactions or the expanding of networks from social media to in-person contexts. Other studies before and after this study’s time frame also found worsening outcomes for young people, including stress and emotional distress (Robillard et al., 2020; Shanahan et al., 2022).

The second most impactful correlate in the data emerged when controlling for age: financial power. Intuitively, the lesser one’s financial power, the greater the depression, hopelessness, anxiety, and sense of control over one’s own life during the pandemic. Participants with personal health vulnerabilities to COVID-19 (e.g., at cardiovascular risk, 65 + years old, immunocompromised) tended to have lesser financial power. This was particularly true for participants endorsing American Indian ethnicity, which was significantly associated with both low financial power and personal health vulnerabilities to COVID-19, but—in a representation of resilience—not distress. American Indian ethnicity was not in itself associated with worse mental health overall, but American Indian participants with personal or community health risks during COVID-19 endorsed significantly greater anxiety and somatic distress than those without. LGBQ-identified participants as well as people with ADA disabilities also had significantly lower financial power than their respective comparison groups and likely related elevations in hopelessness and worry during the pandemic. Disproportionate distribution of resources across groups appears to be connected to the amount of mental health distress experienced (Gibb et al., 2020). Taken together, these data support the role of financial resources in maintaining classic lines of marginalization in the United States, in which future analyses should evaluate as a mediator of the impact of pandemics on mental health and distress.

### Who is most vulnerable to COVID-19?

One-quarter of American Indian participants and nearly one-third of participants with an ADA disability endorsed personal health vulnerabilities that put them at double and triple the risk for COVID-19, compared to the study average overall. In terms of ethnicity, this result may be indicative of the health inequity for American Indians related to historical trauma and oppression (Hatcher et al., 2020). In terms of disability, this result was anticipated due to the literature on health- and immune-related disabilities, as well as the significant loss of services or accommodations for this community during the pandemic (e.g., Jesus et al., 2021). Given their increased need for safety and services during the COVID-19 pandemic, it is unconscionable that 22% of people with disabilities in this study also reported loss of assistance-based services due to COVID-19 pandemic restrictions.

Over one-third of the disabled, LGBQ, and European American subsamples reported having at least one community or family members with health-related vulnerabilities to COVID-19, compared to about one-quarter for all other groups. This may illustrate differences in social network and risk disclosure patterns as well as community risk (Badgett et al., 2020; Salerno et al., 2020a; Signorelli et al., 2020).

Taken together, these data position cismale gender, heterosexual orientation, and non-disabled status as protective factors amidst the COVID-19 pandemic. These results highlight the importance of resource distribution across marginalized populations, particularly as regards the availability or loss of healthcare, employment, and housing in the event of national crises, especially for disabled and GSM populations (Gibb et al., 2020; Salerno et al., 2020b).

In general, about 40% of the entire sample reported living with environmental vulnerabilities to COVID-19 at work and/or home, seen across all ethnic groups in the study. Trend-level findings demonstrating slightly greater rates of environmental vulnerability to COVID-19 among Hispanic/Latinx participants, followed closely by African-American participants, as compared to the other ethnicities in the study. McNicholas and Poydock (2020) suggest it is actually the type of jobs individuals hold that determines their environmental susceptibility to COVID-19 and the literature shows that those who hold minority identities are generally more likely to hold essential positions (Hawkins, 2020).

### Who is most distressed during the COVID-19 pandemic?

#### Demographics and mental health outcomes

Gender was related to the greatest breadth of distress, across all demographic variables explored. Specifically, ciswomen and TGE participants demonstrated greater and more frequent symptoms of depression, worry, somatic complaints, and distress around health fears and uncertainty during the pandemic. This reflects well-established mental health disparities for women and for GSM populations (LGBT Foundation, 2020; Peterson et al., 2020).

Women and gender minorities also reported feeling less in control of their lives. This effect on LOC could reflect the relative privilege cismen experience in the world. In addition, this relative privilege can be connected to better access to resources for healthcare and stability in employment and financials during the pandemic (Landivar et al., 2020), which may help to explain the gender differences in mental health outcomes. When considered dispositionally, preexisting internal LOC may have a significant, positive influence on a person’s ability to cope with stressors as demonstrated in recent COVID-19 pandemic literature (Bachem et al., 2020; Sigurvinsdottir et al., 2020). However, it could be that one mechanism of pandemics’ impact on mental health is a decrease in internal LOC due to situational powerlessness. It may be that during a pandemic, LOC becomes more fluid, incorporating fiscal power, and acting as a moderator of the impact of the pandemic on mental health (Bachem et al., 2020).

Consistent with previous literature (Kamal et al., 2021), the sexual minority participants in this study reported greater levels of hopelessness, worry, and external LOC, in comparison with the heterosexual sample. Exploratory *post hoc* analyses replicated pre-pandemic findings that queer and bisexual individuals often suffer even more than their sexual minority peers (Ross et al., 2018). Our LGBQ sample, like many others (e.g., Gibb et al., 2020), did demonstrate greater rates of poverty than the heterosexual sample. However, findings did not indicate elevated psychological distress for sexual minorities across the board, mirroring theories on unexpectedly strong mental health and resilience among some of the more oppressed ethnicities in the United States (Paul, 2018).

Participants with physical disabilities reported significant but shallow elevations in scores for depression, hopelessness, worry, obsessions/compulsions, and panic, as well as both clinically and statistically significant elevations in pain/somatic symptoms, compared to non-disabled participants. First, these vulnerabilities and distress within the disabled sample may be related to its greater rate of income at or below the poverty line, as compared to the non-disabled sample. Financial resources may serve to insulate some, but not all, people with disabilities from the impact of the COVID-19 pandemic. Furthermore, this high percentage of people who are disabled below or at the poverty line (60%) indicates that they are likely to have government-based health insurance or assistance. Finally, the disruption of disability assistance and services by shelter-in-place policies paired with the use of medical rationing illustrates how regulations by governing bodies to combat COVID-19 may introduce or compound health risks, pain, uncertainty, and fear for the disabled community (Boyle et al., 2020; Lund and Ayers, 2020). Previous literature corroborates that loss of services across disabled populations is commonly associated with increases in injury, pain, and psychological distress (Jesus et al., 2021), as was the case for half of our sample with ADA disabilities. Overall, these results highlight both the deleterious consequences of the loss or lack of essential provisions, as well as the importance of ensuring sustained, unbiased, and consistent accommodations, medical supplies, and services for all disabled populations.

Although our data indicate that many people with disabilities have statistically significant mental health distress, in five areas it is indistinguishable from distress levels of non-disabled participants. One possible interpretation is a ceiling effect, in which the amount of pre-pandemic distress for oppressed populations is already so great that the impact of COVID-19 caused milder elevations for them than for others (e.g., Llera and Newman, 2010). Another compelling theory is the resiliency interpretation, in which a history of life experiences leads to a greater resilience even in the face of greater stress, resulting in score averages similar to more privileged populations, who may nonetheless be experiencing fewer stressors overall (e.g., Fishback et al., 2020).

In terms of ethnicity, it was the European American group that endorsed significantly greater scores in depression and worry than all other ethnicity samples, particularly the African-American and Hispanic/Latinx samples. No other mental health score means were found to differ significantly by ethnicity. While this disproves our original hypothesis, it is in line with recent COVID-19 research (Graham et al., 2020; Liu et al., 2020), again explained by some sociologists as a feature of resilience against long-standing oppression, in which low levels of omnipresent stress protect some people against the spikes in stress that the COVID-19 pandemic caused for others (Llera and Newman, 2010; Fishback et al., 2020). This theory is supported by the positive correlation between African-American ethnicity and internal LOC, particularly as it relates to resilience in mental health (Bachem et al., 2020). Alternately, the present data could reflect some underreporting of distress by researchers or ethnic minority groups seen in both pandemic and non-pandemic-related contexts (Rochon et al., 2004; Raghav et al., 2021). However, even commensurate rates of distress among all ethnicities would suggest great resilience among the African-American community, given the proven medical disparities and disproportionate impact of COVID-19 on the African-American community (Raine et al., 2020).

Each ethnicity had slightly different COVID-19 risk factors associated, on average, greater with depression, anxiety, or pandemic-related distress: Among African Americans, it was having family and/or community members vulnerable to COVID-19, for American Indians, it was endorsing personal health-related vulnerabilities to COVID-19, or living in urban areas, among Asian Americans, it was being in a home or work with greater environmental COVID-19 risk, and among Hispanic/Latinx participants, personal health-related vulnerabilities to COVID-19 were related only to depressive symptomology. This result may be explained by underreporting in mental health (Rochon et al., 2004), or else because factors beyond the scope of these analyses are determining the mental health of Hispanic/Latinx people during COVID-19. European Americans living in urban areas or under strict shelter-in-place policies reported, on average, greater distress around feeling restricted and disconnected, greater internal LOC, and increased hope. Interestingly, the variability in scores among the European American sample limited its usefulness as a cohesive category, perhaps due to intersectionality with other identities.

There are limitations to this study. First, the data collected are cross-sectional in nature and represent a short 2-month period of time during the COVID-19 pandemic in the United States, which limits the ability to generalize further and also to justify causal relationships. Although quota convenience sampling was utilized to obtain a diverse sample, there were still limitations in the ability to examine intersectional identities and very limited ability to examine sexual orientation and gender outside of exploratory analysis. It is important to consider the limitations of this study with reference to the impact of COVID-19 on participants’ finances as this difference is not fully captured within the present study; other researchers have found that anticipation of financial struggles was associated with greater anticipated mental health struggles (Piltch-Loeb et al., 2021). Future work in these areas could use larger samples with nested stratification to further explore intersectionality effects between ethnicity, gender, disability, and sexual orientation. Although our TGE was quite small, exploratory analyses revealed their rates of vulnerability were high comparatively to their cisgender peers; research conducted during a similar time frame to this study with a large TGE population indicated pandemic exacerbation of existing mental health struggles (Kidd et al., 2021). Further research is needed to examine the experiences of the TGE population as differentiated from cisgender women and other sexual orientations as differentiated from the monolith of LGBQ.

## Conclusion

Data from this study were collected during the COVID-19 pandemic and in the midst of the significant nationwide protests for racial justice and the continued growth of the Black Lives Matter movement. The present data are a snapshot of that specific time frame, in which various stages of shelter-in-place were in effect in many places, a pattern which may again become relevant during the emergence of future COVID-19 variants or pandemics with restrictive protective measures. Furthermore, this study highlights differential impact of COVID-19 across marginalized identities. Our study did not aim to examine the influences of privilege and intersectional identities, but reaped results that suggest the influence of these phenomena and the intention was to examine results within the context of sociopolitical oppression experienced by marginalized groups. Age was an extremely influential factor throughout our analyses and should be considered or controlled for in all pandemic-related research. In some instances, relative privilege appeared to be a protective factor against psychological distress. As with our Hispanic/Latinx, African-American, ADA disability, and LGBQ samples, the potential of ceiling effects and resilience to stress may render lower rates of mental health disorder in response to equivalent stressors as socially and financially privileged communities and hence understate the impact of the COVID-19 pandemic on marginalized populations.

Taken together, our study has many implications within clinical work and beyond. Disparities in mental health outcomes and COVID-19 risks exist among marginalized communities, with unique experiences across demographic factors, and it is our hope that these data may be utilized by governing bodies and clinicians as the repercussions of the COVID-19 pandemic continue to develop, and as global planning efforts evolve for future pandemics. Evidence is in line with the theory that most mental health disparities during the pandemic are amplifications of preexisting social disparities, impacting far more people than those at greatest risk of mortality to COVID-19. Government and other organizations must take into consideration the unique needs of populations regarding regulations in the face of the COVID-19 pandemic and other national or global crises and inquire directly with these populations in regard to their needs instead of prescribing interventions from a top–down approach. Our findings show the importance of acknowledging resiliency in marginalized populations, instead of seeing the disparity in distress as an assumed experience. Ultimately, not everyone is impacted equally by the COVID-19 pandemic, and it is critical to examine these unique differences.

## Data availability statement

The datasets presented in this article are not readily available because the authors have future planned publications at this time. Requests to access the datasets should be directed to the JB, jbrooks4@alliant.edu.

## Ethics statement

This study was approved by the Alliant International University Institutional Review Board (IRB). Participants provided informed consent electronically.

## Author contributions

All authors listed have made a substantial, direct, and intellectual contribution to the work, and approved it for publication.
